# Value of routine blood count surveillance in detecting relapse in acute lymphoblastic leukemia

**DOI:** 10.3389/fped.2024.1488686

**Published:** 2025-01-06

**Authors:** Sarah AlHarbi, Areej Taha, Ahmed Ashi, Naglla Elimam, Sami Althubaiti

**Affiliations:** ^1^ Department of Pediatric Oncology, King Abdullah Specialized Children’s Hospital, King Abdulaziz Medical City, Ministry of National Guard Health Affairs, Jeddah, Saudi Arabia; ^2^ King Abdullah International Medical Research Center, King Abdul Aziz Medical City, Jeddah, Saudi Arabia; ^3^ King Saud Bin Abdulaziz University for Health Sciences, Makkah—Jeddah Hwy, King Abdul Aziz Medical City, Jeddah, Saudi Arabia

**Keywords:** acute lymphoblastic leukemia, complete blood count, surveillance, relapse, children

## Abstract

**Background:**

Children with acute lymphoblastic leukemia (ALL) have excellent outcomes, with >85% survival without relapse following contemporary therapies. Clinical and complete blood count (CBC) assessments are commonly used surveillance methods to detect relapses. We aimed to evaluate the efficacy of routine blood testing for detecting relapse using a systematic method of assessing normal and abnormal results.

**Methods:**

This a retrospective, single center study included children aged 1–14 years diagnosed with ALL who completed therapy and were in complete remission. Demographic data, leukemia subtypes, risk stratification, treatment responses, and outcomes were also reviewed. CBC tests were evaluated, and abnormal results were categorized. The relapse groups were classified as asymptomatic and symptomatic relapses. The clinical outcomes of relapse and complications were analyzed. The sensitivity, specificity, positive predictive value, and negative predictive value of surveillance laboratory tests for predicting relapse after the end of treatment were evaluated.

**Results:**

In total, 187 patients underwent 2074 CBC tests. Ten patients underwent full surveillance, whereas the remaining patients underwent partial surveillance. The median number of surveillance blood draws per patient was 12. Relapse was observed in nine patients. Only three patients had asymptomatic relapses. Neutropenia, leukopenia, pancytopenia, thrombocytopenia, and anemia were observed in 98, 89, 10, 6, and 3 patients respectively. The sensitivity and specificity of neutropenia, leukopenia, thrombocytopenia, anemia, and pancytopenia were 11.11% and 47.9%, 0% and 50%, 33.3% and 98.31%, 0% and 99.4%, and 33.3% and 96.07%, respectively. No differences were observed between patients who had asymptomatic relapses and those whose clinical outcomes or consequences had symptomatic relapses.

**Conclusion:**

Relapse after completion of therapy in ALL is rare. Regular blood count surveillance does not predict clinical outcomes or relapse. Prospective studies are required to assess appropriate risk-based surveillance and its effects on patient outcomes and quality of life.

## Introduction

Acute lymphoblastic leukemia (ALL) is the most prevalent childhood cancer, occurring in approximately 25% of all children aged 0–14 years (1). When treated based on risk adaptation using multi-agent chemotherapy protocols, children show remarkable improvements in survival rates, with an overall survival (OS) rate >90% (2).

Relapse is the leading cause of ALL treatment failure, particularly in patients with B-cell ALL. The relapse rate in patients with ALL ranges from 15%–20%, and the OS rate after relapse ranges from 40 to 70%. All relapses can be classified according to the site as isolated bone marrow relapse, extramedullary relapse, or combined bone marrow with extramedullary relapse, whereas timing can range between early (occurring within 18 months of diagnosis) and late (after 36 months) (3). The addition of blinatumomab, a bispecific antibody targeting CD3 and CD19, in patients with high-risk relapsed B-ALL led to better outcome (4, 5).

Only 5%–17% of ALL relapses are asymptomatic, with no difference in survival rates between relapses detected using abnormal cell counts and symptomatic relapses (6, 7).

Data on the sensitivity and specificity of routine blood count surveillance for detecting relapse and its effects on disease outcomes and complications are scarce. We aimed to evaluate the efficacy of routine blood testing using a systematic method of assessing normal and abnormal results.

## Methods

We conducted a retrospective cohort study between 2014 and 2021 in patients diagnosed with ALL aged 1–14 years treated at our institution. For B-cell ALL, our center risk stratifies, treats, and assesses responses according to the Children's Oncology Group (COG) AALL0932 and AALL1131 for standard risk (SR) and high risk (HR), respectively (8, 9). For T-cell ALL, we treat patients according to AALL0434 (10) with modifications (11). Risk and relapse patients were stratified according to COG AALL1331 (4), and patients were identified from the hospital database. All patients with leukemia who had been treated and completed therapy in complete response and were followed up at our institution were included. Patients with Down syndrome or inherited cytopenia were excluded from this study. Data abstracted from electronic health records included patient demographics; leukemia subtype; risk stratification; response and outcome; follow-up laboratory testing results, including white blood cell count, hemoglobin level, platelet count, and absolute neutrophil count; relapse site and time; and post-relapse complications. Follow-up data were collected after primary treatment completion, with the last date of chemotherapy used as the date of completion.

The patients were followed up according to the institutional guidelines, which included clinic visits and laboratory tests every 1 month for the first 6 months, every 2 months for the second 6 months of the first year and every 3–4 months for the second and third years, every 6 months thereafter for the fourth year, and annually thereafter. This study was approved by the institutional review board (No: NRJ21J-205-08).

The patients were classified into full laboratory surveillance (100% adherence to surveillance protocol), partial surveillance (<100% adherence), and no surveillance groups. The relapse groups were classified as asymptomatic (relapse diagnosed using routine blood tests) and symptomatic (diagnosed clinically). The clinical outcomes related to relapse or complications were analyzed for each category.

For each surveillance laboratory component, we assessed whether the test results were normal or abnormal based on the Common Terminology Criteria for Adverse Events version 5.5.

The patients were considered to have abnormal results if their test abnormalities were grade 2 or higher.

## Statistical analyses

Baseline patient characteristics are expressed as absolute frequency (%) and interquartile range (IQR) values. Categorical data were compared using Fisher's exact tests, and continuous data are presented as medians and IQRs and compared using the Mann–Whitney *U* test. The value of blood count in detecting relapse was assessed using sensitivity, specificity, negative predictive value (NPV), and positive predictive value (PPV). Number needed to screen (NNS) is defined as the number of patients that need to be screened for the duration to detect relapse event. Statistical tests were performed using GraphPad Prism version 9.0.0.

## Results

Between January 2014 and December 2021, 187 patients with ALL met the eligibility criteria for this study. The median age of the patients was 12 (range, 10–16) years, and 161 (86.10%) had B-cell ALL. In total, 113 (60.4%) patients had National Cancer Institute Standard-risk (SR). Ninety-three (49.4%) patients were assigned to the SR treatment group, whereas 74 (39%) patients were assigned to the High-risk (HR) group. Most patients [130 (69.5%)] had favorable cytogenetics, and 158 (84.4%) patients attained negative minimal residual disease by the end of induction. No cases of treatment-related leukemia were identified in our cohort (Table 1).

**Table 1:** Patient and disease characteristics.

Baseline characteristic	Measure (%)
The total of eligible patients	187
Median age (y)	12
Sex	
Male	109 (58.29%)
Female	78 (41.71%)
Diagnosis	
B-cell ALL	161 (86.10%)
T-cell ALL	26 (13.90%)
NCI risk stratification	
SR	113 (60.4%)
HR	74 (39.57%)
Treatment assignment	
SR	93 (49.47%)
HR	74 (39.36%)
Upgraded to HR due to positive EOI MRD	18 (9.57%)
Upgraded to HR due to unfavorable cytogenetic	3 (1.60%)
Cytogenetic—molecular	
Favorable	130 (69.52%)
Natural	43 (22.99%)
Unfavorable	7 (3.74%)
Failed	7 (3.74%)
MRD	
Negative	158 (84.49%)
Positive	29 (15.51%)
**Blood counts**	
Total number of CBC	2,074
Median laboratory test per patient (IQR)	12 (7–14)
Surveillance blood count	
Full	9 (4.80%)
Partial	178 (95.19%)
Not complete	00 (00.0%)
CBC abnormality during surveillance	
Neutropenia	98 (47.57%)
Anemia	3 (1.46%)
Thrombocytopenia	6 (2.91%)
Pancytopenia	10 (4.85%)
Leukopenia	89 (43.20%)
Action taken for CBC abnormality	
Observation	96 (80.00%)
Blood smear	22 (18.33%)
Bone marrow aspiration	2 (1.67%)
Relapse	
Yes	9 (4.79%)
No	179 (95.21%)
Detection of relapse	
By symptoms	6 (66.67%)
By routine laboratory	3 (33.33%)
Outcome	
Complete remission	183 (98.92%)
Death	3 (1.08%)
Treatment-related leukemia	0 (0%)

MRD: positive, >0.01%, MRD: negative, <0.01%.

SR, NCI SR (WBC < 50 000–age 1–9.99 years), HR, NCI high risk (WBC > 50 000–age > 10 years).

Favorable cytogenetic ETV6-RUNX1, double trisomy 4 + 10.

Unfavorable cytogenetic: KAMT2A rearrangement, hypodiploidy, IAMp21, *t*(17:19) Philadelphia + ALL, Induction failure M3 BM on day 29.

EOI, end of induction; MRD, minimal residual disease; IQR, interquartile range.

## Disease surveillance

The total complete blood count (CBC) tests performed for the whole cohort were 2074; the median laboratory test per patient was 12 (IQR, 7–14). Laboratory abnormalities were detected in 201 tests; the most common abnormalities detected were neutropenia 98 (47.57%) and leukopenia 89 (43.2%). Most patients [96 (80%)] were observed; 22 (18.3) and 2 (1.6%) patients underwent blood smear and bone marrow assessment, respectively.

Most patients [178 (95.1%)] underwent partial surveillance; nine (4.79%) patients relapsed at a median follow-up period of 18 months. Of the total 187 patients, only three relapses were identified through routine blood tests (Table 1). The calculated number needed to screen (NNS) to detect a relapse was 63.

The most frequent abnormal laboratory results were thrombocytopenia [*n* = 3 (33%)] and pancytopenia [*n* = 3 (33%)]. The median blast percentage at relapse was 70% (IQR, 52%-76%) (Table 2). Comparing the blast percentage between the two groups at initial presentation of relapse, the symptomatic and asymptomatic blast percentages were 57% (IQR, 32%-72%) and 76.5% (IQR, 70%-83%) (*p* = 0.0397), respectively.

**Table 2:** Relapse characteristics.

Relapse patient characteristic	Measure %
The total of relapsed patients	9
Median time to relapse	18 months
Median of blast percentage (IQR)	70% (52%–76%)
Time of relapse	
Early	3 (33.33%)
Late	6 (66.67%)
Site of relapse	
Bone marrow	6 (66.67%)
Extramedullary	1 (11.11%)
Combined	2 (22.22%)
Symptoms of relapse	
Fever	1 (11.11%)
Bone pain	2 (22.22%)
Lymphadenopathy	1 (11.11%)
Others	2 (22.22%)
Asymptomatic	3 (33.33%)
Risk stratification at initial diagnosis	
SR	2 (22.22%)
HR	3 (33.33%)
HR due to positive end of induction MRD	4 (44.44%)
Count at relapse	
Normal	2 (22.22%)
Leukocytosis	0 (0.00%)
Anemia	0 (0.00%)
Thrombocytopenia	3 (33.33%)
Neutropenia	1 (11.11%)
Pancytopenia	3 (33.33%)

Definition according to the AALL1,331 protocol.

Early relapse BM < 36 months, extramedullary < 18 months.

Late relapse BM > 36 months, extramedullary > 18 months.

BM relapse: M3 marrow at any point after achievement remission extramedullary: CNS positive blast and WBC ≥ 5 or clinical sign.

MRD, minimal residual disease positive if <0.01%.

The sensitivity and specificity of neutropenia, leukopenia, thrombocytopenia, anemia, and pancytopenia were 11.11% and 47.9%, 0% and 50%, 33.3% and 98.31%, 0% and 99.4%, and 33.3% and 96.07%, respectively. PPV and NPV were assessed for each abnormal blood count and are summarized in Table 3.

**Table 3:** Measurement of screening accuracy.

Laboratory test	Status	Relapse	No relapse	Sensitivity	Specificity	PPV	NPV
		(*n* = 9)	(*n* = 178)	(95% CI)	(95% CI)	(95% CI)	(95% CI)
Neutropenia	Yes	1	94	11.11	47.19	1.05	91.30
	No	8	84	(0.28–48.25)	(39.68–54.8)	(0.17–6.36)	(88.82–93.28)
Leukopenia	Yes	0	89	0.000	50	0.000	90.82
	No	9	89	(0–33.63)	(42.43–57.7)	(0–4.1)	(89.52–91.97)
Thrombocytopenia	Yes	3	3	33.33	98.31	50.00	94.69
	No	6	175	(7.49–70.07)	(95.15–99.65)	(18.95–81.05)	(94.84–97.89)
Anemia	Yes	0	1	0.00	99.4	0	95.16
	No	9	177	(0.00–33.63)	(96.8–99)		(95.11–95.21)
Pancytopenia	Yes	3	7	33.33	96.07	30.00	96.61
	No	6	171	(7.49–70.07)	(92.07–98.4)	(11.69–58.12)	(94.72–97.84)

PPV, positive predictive value; NPV, negative predictive value; CI, confidence interval.

## Relapse characteristics and outcomes

Out of the nine patients with relapse, 66.6% (*n* = 6) had late relapse. Bone marrow relapse was the most common relapse (66.6%), and most relapse occurred in the high-risk group [*n* = 7 (77.7%)]. No predominant symptoms were associated with symptomatic relapse.

Treatment-related complications, including infection, tumor lysis syndrome, and medication toxicity, were comparable between the two groups (Table 4).

**Table 4:** Relapse treatment and outcomes.

Relapse characteristics	Symptomatic	Asymptomatic	Fisher’s exact test
	*N* (%)	*N* (%)	
Treatment of relapse			
Chemotherapy	3 (60%)	3 (75%)	>0.9999
HSCT	2 (40%)	1 (25%)	>0.9999
Complication during reinduction			
Yes	4 (80%)	3 (75%)	0.5238
No	1 (20%)	1 (25%)	>0.9999
Blast (median)	57%	76.5%	0.0397^a^
Types of complication during reinduction			
Prolonged fever	3 (60%)	1 (25%)	>0.9999
Asparaginase allergy	1 (20%)	1 (25%)	0.4444
Steroid complication	1 (20%)	1 (25%)	>0.9999
Renal complication	0 (0%)	0 (0%)	>0.9999
TLS	0 (0%)	0 (0%)	>0.9999
Outcome			
Complete remission	4 (80%)	3 (75%)	>0.9999
Death	1 (20%)	1 (25%)	>0.9999

Complete remission is defined according to the AALL1331 protocol as M1 marrow with no evidence of a blast with peripheral count recovery.

HSCT, hematopoietic stem cell transplantation; TLS, tumor lysis syndrome.

^a^ Mann–Whitney *U* test.

Seven patients achieved remission after reinduction therapy, and three patients underwent hematopoietic stem cell transplantation. Seven patients were alive at the median follow-up period of 30 (IQR, 12–36) months (Table 4).

## Discussion

Treatment outcomes of children with ALL have improved over the last few years (2). An ideal surveillance protocol should encompass modalities capable of detecting most cancer types commonly found in at-risk individuals. Additionally, the selected modalities should demonstrate effectiveness in the early detection of asymptomatic tumors that can be successfully managed with minimal treatment-related morbidities. Moreover, surveillance protocols must be assessed to demonstrate that early detection of asymptomatic tumors ultimately leads to improved overall patient survival (12). Several attempts to identify relapsed acute lymphoblastic leukemia earlier through surveillance failed to show its effects on outcomes (6, 7, 13). The outcomes of patients with relapse largely rely on disease phenotype, timing of relapse, sites of relapse, and response to therapy (3). It is imperative to assess whether the surveillance tool used to identify relapse is sensitive and specific and can detect disease at low burden, enhance outcomes, and minimize complications. Our data showed that relapse was uncommon after therapy completion. Only nine of our patients relapsed after treatment completion. Our patient population underwent a large number of post-treatment tests, with an average of 12 tests per patient (range, 7–14), but only three patients showed evidence of suspected relapse based on blood count, no patient developed treatment-related leukemia, and in patients with non-relapse, or persistent cytopenia required intervention.

In our cohort, 39% of patients were classified as high risk indicating a significant proportion at risk of relapse.

Abnormal blood counts following therapy for acute lymphoblastic leukemia (ALL) were prevalent, with 57% of our patients' exhibiting abnormalities in one or more CBC tests. The sensitivity, specificity, and positive predictive value (PPV) of the blood tests were low. However, the negative predictive values, were high ranging from 90.0%–96.6% across different CBC parameters, underscores the reliability of CBC as low-risk method for ruling out relapses. Nevertheless, the high likelihood of benign abnormal results may substantially impact the quality of life (QoL) for patients and their families, contributing to increased anxiety and prompting additional testing. No differences were observed in treatment-related complications or responses. We observed a slightly higher blast percentage in asymptomatic relapse cases than in symptomatic relapse cases. This observation may indicate the presence of subtle symptoms and signs that were not readily apparent to either the family or treating physician. This study highlights the necessity for a balanced approach to surveillance that weighs the benefits of early detection against the psychological effects of frequent testing for both high-risk and standard-risk groups. Our study highlights the limitations of routine blood testing for detecting relapse in children with ALL and underscores the importance of developing more sensitive and specific diagnostic methods. The comparable clinical outcomes and treatment-related complications between symptomatic and asymptomatic relapse cases challenge the need for post-treatment surveillance. Furthermore, considering the limitations of routine blood testing in detecting relapse, an important question arises as to whether clinical judgment alone can replace laboratory assessment in relapse surveillance. Our study has some limitations, including the inherent limitations of retrospective studies, a small sample size, and the fact that the effects of such extensive surveillance on QoL was not evaluated. A prospective study evaluating the effectiveness of clinical assessment as a stand-alone approach versus a combined approach involving laboratory assessment and evaluate their effects on patients' QoL, with the aim of enhancing the post-treatment evaluation and management of children with ALL.

## Data Availability

The original contributions presented in the study are included in the article/Supplementary Material, further inquiries can be directed to the corresponding author.
